# Supplementary material for: Beta-Arrestin 1 Deficiency Enhances Host Anti-Myeloma Immunity Through T Cell Activation and Checkpoint Modulation
Source: Int J Mol Sci. 2025 Nov 27;26(23):11478. doi: 10.3390/ijms262311478 (PMC12692176; doi:10.3390/ijms262311478)

Beta-arrestin 1 deficiency enhances host anti-myeloma immunity through T cell activation and checkpoint modulation

Jian Wu[1§], Xiaobei Wang[1§], Shaima Jabbar[1], Niyant Ganesh[2], Emily Chu[3], Vivek Thumbigere Math[2], Lindsay Rein[1], and Yubin Kang[1*]

Supplementary materials

The gating strategies: (1) FSC/SSC gating for live cells, (2) singlet discrimination, (3) lineage-specific markers for T cells (CD3+CD4+ and CD3+CD8+), and (4) myeloid populations (CD11b+Ly6C+Ly6G- for M-MDSCs).

Supplementary Figure S1: Gating strategies for T cells, B cells and NK cells

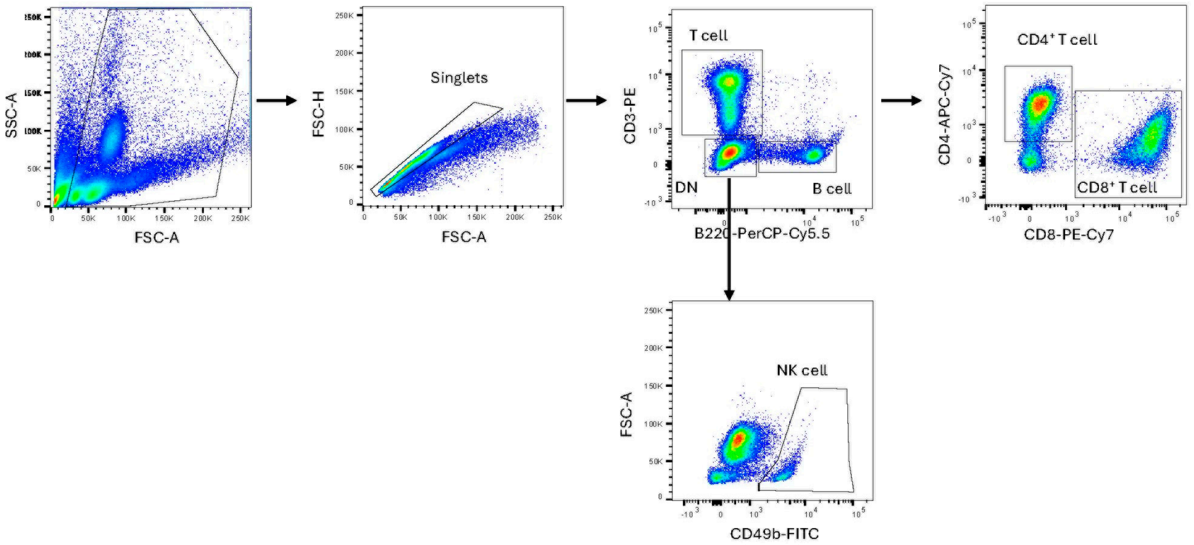

Supplementary Figure S2: Gating strategies for MDSCs

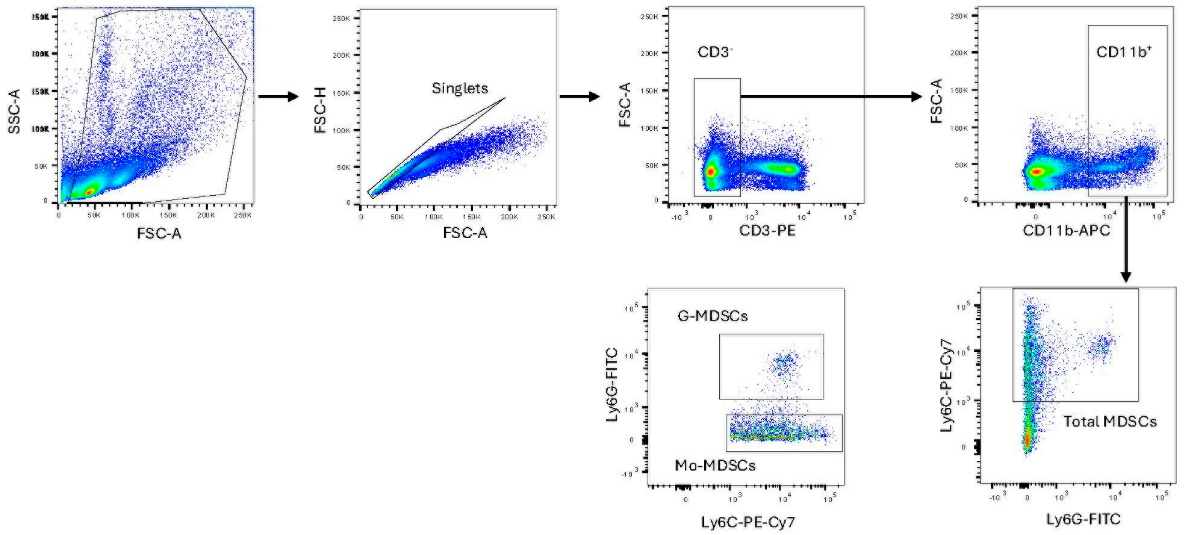

Supplement: Supplementary file 1 [file ijms-26-11478-s001.zip › ijms-3811985-supplementary.pdf]
